# Assessment of the prognostic role of neutrophil-to-lymphocyte ratio following complete resection of thymoma

**DOI:** 10.1186/s13019-018-0805-7

**Published:** 2018-11-19

**Authors:** Piergiorgio Muriana, Angelo Carretta, Paola Ciriaco, Alessandro Bandiera, Giampiero Negri

**Affiliations:** Department of Thoracic Surgery, San Raffaele Scientific Institute, Milan, Italy

**Keywords:** Neutrophil-to-lymphocyte ratio, Thymoma, Surgery, Prognostic markers, TNM staging

## Abstract

**Background:**

The introduction of the new TNM staging system for thymic epithelial malignancies produced a significant increase in the proportion of patients with stage I disease. The identification of new prognostic factors could help to select patients for adjuvant therapies based on their risk of recurrence. Neutrophil-to-lymphocyte ratio (NLR) has recently gained popularity as reliable prognostic biomarker in many different solid tumors. The aim of this study is to assess the utility of NLR evaluation as a prognostic marker in patients with surgically-treated thymoma.

**Methods:**

A retrospective analysis was conducted among patients who underwent resection for thymoma in a single center. Patients were divided in two groups, under (low-NLR-Group = 47 patients, 60%) and above (high-NLR-Group = 32 patients, 40%) a ROC-derived NLR cut-off (2.27). Associations with clinical-pathological variables were analyzed; disease-free survival (DFS) was identified as the primary endpoint.

**Results:**

Between 2007 and 2017, 79 patients had surgery for thymoma. Overall 5-year DFS was 80%. Univariate survival analysis demonstrated that NLR was significantly related to DFS when patients were stratified for TNM stage (*p* = 0.043). Five-year DFS in the low-NLR-Group and in the high-NLR-Group were respectively 100 and 84% in stage I-II, and 66 and 0% in stage III. TNM stage resulted as the only independent prognostic factor at multivariate analysis, with hazard ratio of 3.986 (95% CI 1.644–9.665, *p* = 0.002).

**Conclusions:**

High preoperative NLR seems to be associated to a shorter DFS in patients submitted to surgery for thymoma and stratified for TNM stage.

## Background

Thymoma is the most common primary tumor of the anterior mediastinum, with a reported incidence of 1.7 cases per million per year in Europe [1]. Complete surgical resection is the mainstay of treatment [1, 2], and is associated with favorable survival results. Nevertheless, recurrence after surgery is observed in up to 50% of patients with locally-advanced disease [2] and can occur even many years after initial treatment due to the indolent nature of these slowly-progressive tumors. Recurrence has been related to many prognostic factors, such as age, tumor stage, size, World Health Organization (WHO) histological classification, completeness of resection, presence of paraneoplastic syndromes, local invasion and presence of lymph-nodes metastasis [1, 2]. Furthermore, genomic factors may also influence prognosis in patients with thymoma [3].

Following the proposals of a working group of the International Thymic Malignancies Interest Group (ITMIG) and the International Association for the Study of Lung Cancer (IASLC), a new staging system for thymic epithelial tumors has been included in the last TNM cancer staging system revision [4, 5]. TNM staging has been shown to have a stronger correlation with disease-free survival (DFS) analysis in comparison with the Masaoka-Koga staging system [6]. However, following further analysis, the introduction of the new TNM staging system for thymic epithelial malignancies produced a significant imbalance of tumors toward stage I [6]. The identification of new prognostic factors may therefore be useful to obtain an additional stratification of early-stage tumors according to the risk of recurrence to define the indications for adjuvant treatments.

Recently, a growing number of studies examined the role of tumor-induced systemic inflammation. Neutrophils cover an important role in the tumor microenvironment. These cells are able to produce cytokines and oxidative stress derivatives, and may favor tumor promotion, progression and distant spread by inhibiting the antitumor activity of the immune system [7, 8]. Among inflammatory markers, the prognostic significance of neutrophil-to-lymphocyte ratio (NLR) has been extensively investigated in the very last few years. Many studies demonstrated that high NLR value is predictive of reduced overall survival (OS), cancer-related survival, DFS, progression-free survival, and enhanced resistance to therapies in a multitude of solid neoplasms [7, 9]. NLR could therefore represent an easily available and cost-effective indicator of tumor-related inflammatory response for therapeutic planning and follow-up.

The aim of our study is to investigate the prognostic role of NLR and its association to clinical and pathological characteristics in patients with thymoma who underwent surgical resection at our Department.

## Methods

The data of patients who underwent complete surgical resection for pathologically proven thymic epithelial tumor at our Department of Thoracic Surgery of the San Raffaele Scientific Institute, Milan, between January 2007 and April 2017 were retrospectively analyzed. Exclusion criteria included thymic carcinoma and incomplete resection (R1 or R2).

The following data were entered in a prospective database: age, gender, presence of myasthenia gravis (MG) and MGFA classification, neoadjuvant or adjuvant therapy, surgical approach, tumor histology according to the current WHO classification [10], Masaoka-Koga staging [11], DFS, OS, and cause of death. Histopathologic reports were retrospectively reviewed, and all the cases were reclassified according to the 8th edition of TNM staging [5].

Patients were followed up at intervals of 3 months for the first year after surgery, then every 6 months for the next 2 years, and annually thereafter. Recurrence was defined either in case of histologically proven disease relapse, or in case of radiological evidence of recurrence followed by response to treatment. OS was defined as the time from the date of surgery to death from any cause or the last follow-up. DFS was defined as the time from the date of surgery to recurrence or the last follow-up.

For each patient, a peripheral blood sample was collected between 72 and 24 h before surgery. Complete blood cell count was performed: total white blood cell (WBC), neutrophil, and lymphocyte counts were entered in the database. MG cases who received specific preoperative pharmacologic treatment and patients who underwent induction treatment were not excluded from the analysis. In fact, NLR mean value in these patients did not significantly differ from patients without MG history and patients who did not receive neoadjuvant therapies (*p* = 0.13 and *p* = 0.35, respectively).

**Fig. 1:**
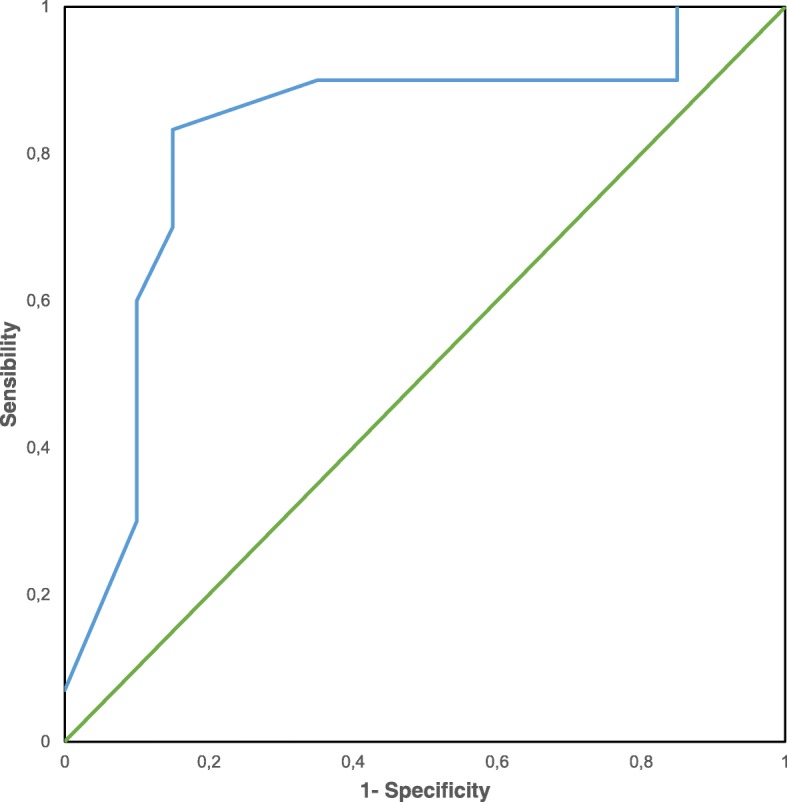
ROC analysis of the preoperative NLR value in the cohort of patients surgically treated for thymoma

Optimal cut-off level for NLR (2.27, sensitivity = 83.3%, specificity = 93.2%) was determined by receiver-operating curve (ROC) analysis (AUC = 0.83, 95% CI 0.73–0.91, Fig. 1); population was divided in 2 groups (above and under the cut-off level), in order to evaluate association with clinical and pathological features, and prognostic value.

Preoperative work-up included routine physical examination, contrast-enhanced chest CT scan, and total body 18-FDG positron emission tomography (PET) scan. The patients who were not already known to be myasthenic before admission underwent a neurological evaluation in order to rule-out the presence of MG.

Preoperative histological diagnosis was obtained in case of indication to neoadjuvant therapy or when differential diagnosis with lymphoproliferative disorder or other anterior mediastinal neoplasms was needed.

Surgery was performed by 5 experienced surgeons by means of either median sternotomy, muscle-sparing thoracotomy or VATS based on size, local extension and location of the tumor. VATS thymectomy has been performed since 2011 in case of small encapsulated tumors (< 2 cm) in non-myasthenic patients. Complete thymectomy (removal of the thymus with surrounding adipose tissue) or extended thymectomy (additional removal of all the mediastinal and pericardiophrenic fat between bilateral mediastinal pleura) was carried out according to MG status, as previously described [12].

### Statistical analysis

Analysis was performed by SAS v9 software (SPSS, v. 18, INC. Chicago, IL, USA). Values are expressed as means ± standard deviation unless otherwise stated. Categorical variables among the groups of patients were compared by means of either Chi-square test or Fisher Exact test as appropriate. Student’s t-test was used to determine the significance of the differences between continuous variables.

Survival curves were estimated by the Kaplan and Meier method. Cox regression analysis was used to assess the risks of the variables. Survival rates of patients grouped according to selected variables were compared by means of the log-rank test. According to the indolent nature of the disease, DFS (i.e. the interval between the date of surgery and the first recurrence) was identified as primary endpoint.

On the univariate survival analysis basis, in order to evaluate the independent contribution of the variables on DFS and OS, a multivariate analysis was performed using the Cox regression method.

Hazard Ratio (HR) and 95% Confidence Interval (95% CI) are shown; a *p*-value < 0.05 was considered statistically significant.

## Results

Between 2007 and 2017, 79 patients underwent complete surgical treatment for pathologically proven thymoma (Table 1). The median follow-up was 29 months (range 1–99 months). At the time of last follow-up, 67 patients (85%) were alive with no evidence of disease, 5 (6%) were alive with disease recurrence (Table 2), and 6 (8%) died of causes other than thymoma relapse (one of acute coronary disease, 4 of metastatic spread of other malignancies not present at the time of surgery, and one for unknown reasons with last follow-up CT scan negative for recurrence). One patient showed disease recurrence 24 months after surgery, and eventually died for pulmonary thromboembolism 75 months after surgery.

**Table 1:** Patients’ characteristics

Total	79
Age (years)	
Mean ± SD; range	58.9 ± 13.4; 27–84
Median	61
Gender (male)	47 (60%)
Myasthenia gravis	15 (19%)
MGFA class I	13
MGFA class IIa	1
MGFA class IIb	1
Neoadjuvant therapy	3 (4%)
Chemotherapy	2
Chemo-radiotherapy	1
Adjuvant radiotherapy	47 (60%)
Surgical approach	
Median sternotomy	65 (82%)
Thoracotomy	4 (5%)
VATS	10 (13%)
Surgical procedure	
Complete thymectomy	64 (81%)
Extended thymectomy	15 (19%)
WHO classification	
A	5 (6%)
AB	32 (41%)
B1	16 (20%)
B2	11 (14%)
B3	15 (19%)
Masaoka-Koga stage	
I	21 (27%)
II	41 (52%)
III	16 (20%)
IV	1 (1%)
TNM stage	
I	65 (82%)
II	2 (3%)
IIIA	11 (14%)
IIIB	1 (1%)

**Table 2:** Clinical and pathological features of the patients who experienced recurrence

Pt #	Age	Sex	MG	NLR	NT	WHO	Masaoka	TNM	AT	DFS	Site	OS	LFU
14	36	M	yes	2.16	no	B3	III	IIIA	yes	27	LR	31	AL
15	63	M	no	5.85	no	B3	III	IIIB	yes	16	LR	47	AL
32	50	M	no	1.68	yes	B2	IV	IIIA	yes	48	LR	61	AL
39	36	M	no	2.27	no	B2	III	I	yes	48	LR	99	AL
50	72	M	no	2.46	no	B3	II	I	yes	24	LR	75	D
66	52	F	no	2.61	yes	B2	III	IIIA	yes	57	LR	59	AL

Legend: *NT* Neoadjuvant therapy, *AT* Adjuvant therapy, *LR* Loco-regional recurrence, *LFU* Status at the time of the last follow up, *AL* Alive, *D* Deceased

According to the IASLC/ITMIG TNM staging, all Masaoka-Koga stage I and II patients and three stage III patients were re-classified as TNM stage I (82% of total patients), and 11 (14%) were classified in TNM stage IIIA. Fifty-two out of 65 patients (80%) in TNM stage I had WHO type A to B1 thymoma, while 10 out of 11 patients (91%) in stage IIIA had WHO type B2 or B3 tumors (*p* < 0.001).

All the three patients who received neoadjuvant therapy before surgery had TNM stage IIIA disease (*p* < 0.001). Forty-seven patients (60%) with locally-advanced disease underwent adjuvant radiotherapy after surgery (45–50 Gy); 96% of them had stage II and III tumors according to the Masaoka-Koga staging system (p < 0.001). No correlation was found between indication to adjuvant treatment and TNM staging (*p* = 0.16).

Overall 1-, 2- and 5-year survival rates for the entire cohort were 100, 94 and 87%, respectively. DFS at 1, 2 and 5 years was respectively 100, 96 and 80%.

Patients were divided in two groups according to the NLR cut-off value (Table 3). Forty-seven patients (60%) had a NLR < 2.27 (low-NLR-Group), and other 32 patients (40%) had a NLR ≥2.27 (high-NLR-Group).

**Table 3:** Classification of patients grouped by NLR < 2.27 (low-NLR-Group) and NLR ≥2.27 (high-NLR-Group)

	Low-NLR-Group (*n* = 47)	High-NLR-Group (*n* = 32)	*P*-value
Age (years)			
< 61	28 (60%)	10 (31%)	0.021*
≥ 61	19 (40%)	22 (69%)	
Gender (male)	26 (55%)	21 (66%)	0.48
Myasthenia gravis	8 (17%)	7 (22%)	0.77
WBC (× 10^9^/L)	7.0	8.1	0.09
Neoadjuvant therapy	2 (4%)	1 (3%)	0.79
Adjuvant radiotherapy	29 (62%)	18 (56%)	0.65
WHO classification			
A	3 (6%)	2 (6%)	0.39
AB	15 (32%)	17 (53%)	
B1	12 (26%)	4 (13%)	
B2	7 (15%)	4 (13%)	
B3	10 (21%)	5 (15%)	
Masaoka-Koga stage			
I	12 (26%)	9 (28%)	0.68
II	23 (49%)	18 (56%)	
III	11 (23%)	5 (16%)	
IV	1 (2%)	0 (0%)	
TNM stage			
I	37 (79%)	28 (88%)	0.028*
II	0 (0%)	2 (6%)	
IIIA	10 (21%)	1 (3%)	
IIIB	0 (0%)	1 (3%)	

Significant data are marked (*)

The proportion of patients older than the median age (61 years) was significantly higher (*p* = 0.021) in the high-NLR-Group (69%) than in the low-NLR-Group (40%). There were no differences between the two groups regarding sex, presence of MG, preoperative WBC count, indication to neoadjuvant and adjuvant therapies, histology and Masaoka-Koga staging.

A significant imbalance emerged in the distribution of patients among TNM stages. In particular, 21% of the low-NLR-Group patients had IIIA/B stage disease; on the other hand, only two patients (6%) in the high-NLR-Group had a thymoma in these stages of the disease (*p* = 0.028).

**Fig. 2:**
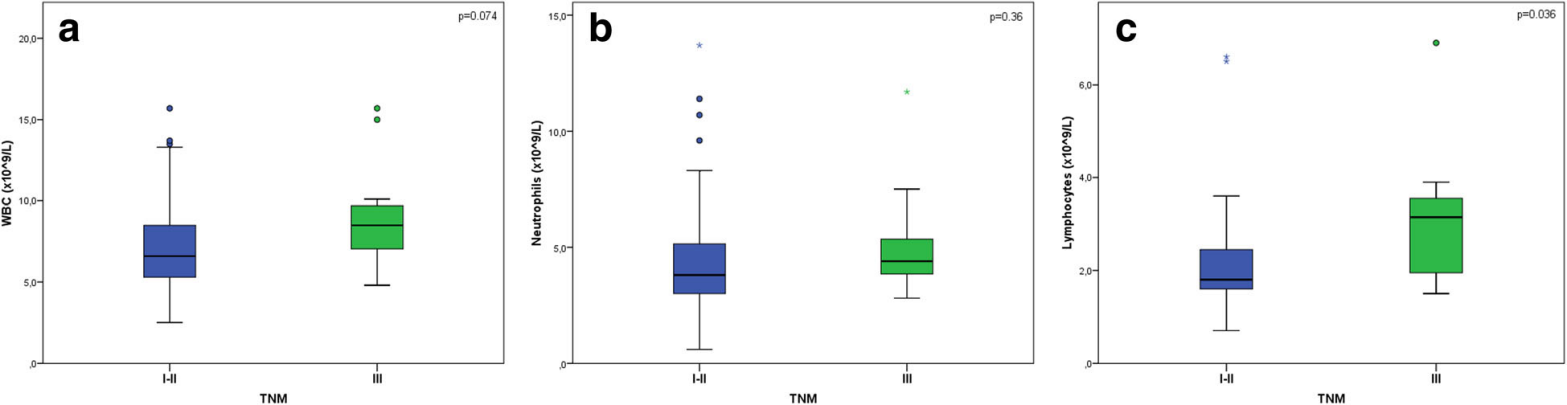
Boxplots reporting WBC, neutrophil and lymphocyte values according to TNM stage. Total WBC (**a**) and neutrophil (**b**) count means did not significantly differ between patients in stages I-II and those in stages IIIA/B (7.2 vs 9.1 × 10^9^/L and 4.4 vs 5.1 × 10^9^/L, *p* = 0.074 and *p* = 0.36, respectively). By contrast, lymphocyte count mean (**c**) resulted higher in stages IIIA/B compared to stages I-II (3.1 vs 2.1 × 10^9^/L, *p* = 0.036)

Total WBC and neutrophil mean counts (Fig. 2a-b) did not differ between TNM stages I-II and stages IIIA/B patients (*p* = 0.074 and *p* = 0.36, respectively). Conversely, mean lymphocyte count in stages I-II and in stages IIIA/B (Fig. 2c) were respectively 2.1 ×  10^9^/L and 3.1 × 10^9^/L (*p* = 0.036), a point which could justify lower NLR values in a higher proportion of stage III tumors.

At univariate survival analysis, WHO classification was the only variable significantly associated with both OS and DFS. AB type thymomas showed the worst 5-year OS (84%, *p* = 0.042), while patients affected by B2 type disease had 40% 5-year DFS (*p* = 0.011). History of neoadjuvant or adjuvant therapy, higher Masaoka-Koga and TNM staging were all significantly associated with a lower DFS (*p* = 0.047, *p* = 0.043, *p* = 0.013, and *p* < 0.001, respectively).

**Fig. 3:**
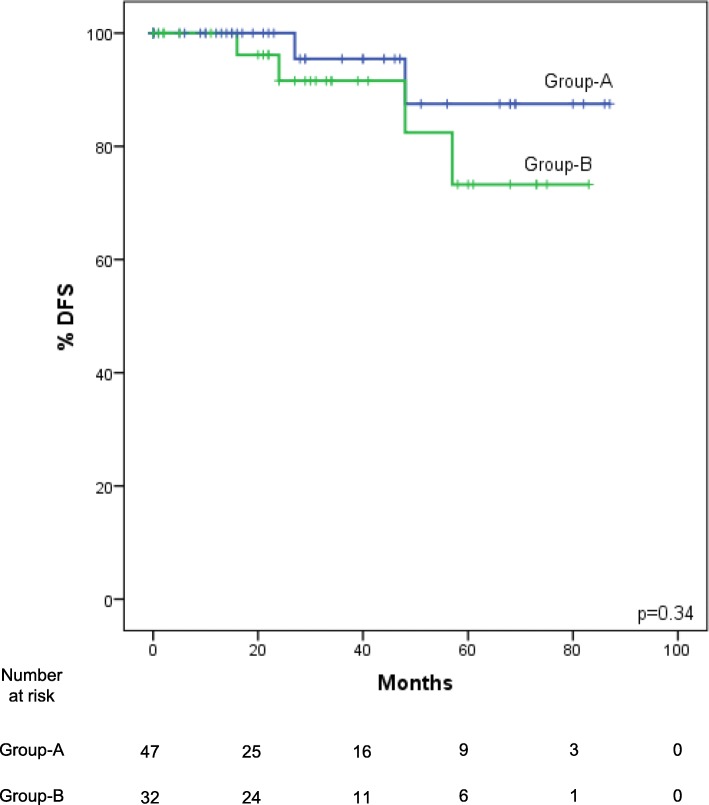
Kaplan-Meier DFS curves for the low-NLR-Group (NLR < 2.27) and the high-NLR-Group (NLR ≥2.27). DFS is lower in the high-NLR-Group patients, but does not reach statistical significance (*p* = 0.34)

**Fig. 4:**
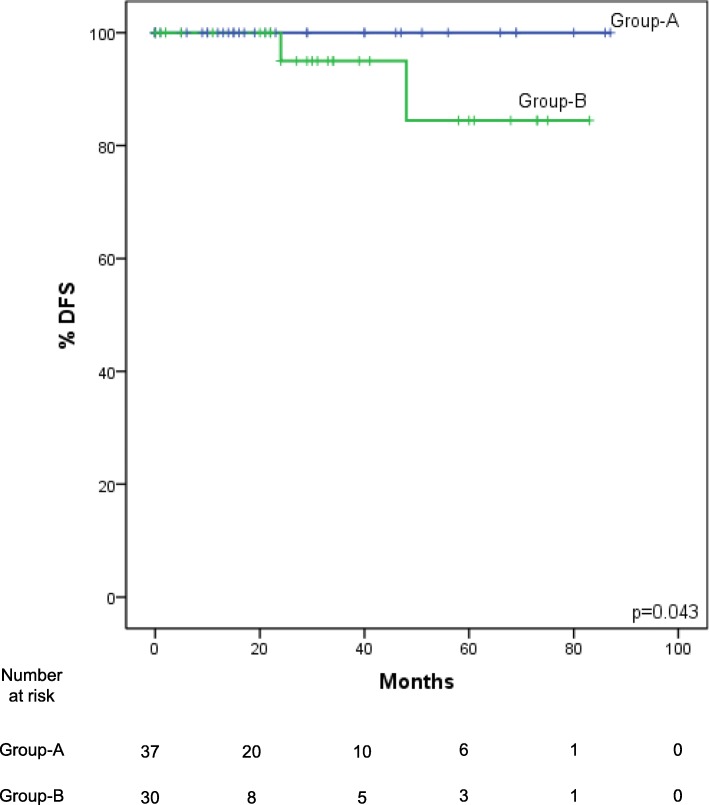
Kaplan-Meier DFS curves for the low-NLR-Group and the high-NLR-Group in TNM I-II stages. None of the patients in the low-NLR-Group had disease relapse. One-, 2- and 5-year DFS in the high-NLR-Group were 100, 95 and 84%, respectively (*p* = 0.043)

1, 2- and 5-year DFS (Fig. 3) was respectively 100, 100 and 88% in the low-NLR-Group, and 100, 92 and 73% in the high-NLR-Group, but these data failed to reach statistical significance (*p* = 0.34). OS was also not significantly different between the low-NLR-Group and the high-NLR-Group (*p* = 0.29). However, following stratification of the patients according to TNM stage, DFS rates for patients in the low-NLR-Group were significantly higher (p = 0.043) than those in the high-NLR-Group both in I-II stages (Fig. 4) and in IIIA/B stages.

At multivariate analysis (Table 4), only TNM staging was an independent prognostic factor for DFS, with a HR of 3.986 (95% CI 1.644–9.665, *p* = 0.002), while NLR approached but failed to reach statistical significance (*p* = 0.066).

**Table 4:** Cox regression multivariate analysis of predictors of DFS

	HR (95% CI)	*P*-value
WHO classification	5.315 (0.812–34.788)	0.081
Masaoka-Koga stage	4.040 (0.366–44.596)	0.254
TNM stage	3.986 (1.644–9.665)	0.002*
NLR	5.272 (0.897–30.995)	0.066

Significant data are marked (*)

## Discussion

In this study, we analyzed the prognostic value of NLR in a group of 79 patients with surgically-treated thymomas. Factors as gender, presence of MG, total WBC count, neo- and adjuvant therapies, WHO classification and Masaoka-Koga staging did not correlate with NLR.

It is noteworthy that a lower proportion of patients with locally-advanced disease (TNM stages IIIA/B) showed a NLR higher than the cut-off value than those in stages I and II (6% vs 21%, *p* = 0.028). At first glance, this result appears in contrast with the data of the literature, since previous reports show that NLR values usually increase along with the invasiveness of the tumor [9]. However, we found that while mean neutrophil count was homogeneously distributed among different stages of the disease, TNM stages IIIA/B patients exhibited a higher mean lymphocyte count (*p* = 0.036), a point which could explain a mean lower NLR in stage III tumors. In detail, since NLR is the quotient between the peripheral neutrophil and lymphocyte counts, a lower NLR value in locally advanced disease could be due to a relative increase of lymphocyte count rather than by a reduction of the neutrophil count.

In fact, thymomas generate autoreactive T-lymphocytes that are responsible for the development of associated paraneoplastic autoimmune diseases [1, 2]. Most of these cells undergo apoptosis before relapse in the systemic blood flow, thus patients affected by thymoma usually show normal peripheral lymphocyte count when compared to healthy controls [13]. However, some authors [14–20] described sporadic patients affected by aggressive thymic malignancies showing absolute peripheral polyclonal lymphocytosis. The distinctive feature of these tumors was an invasive pattern, with local extracapsular infiltration of mediastinal fat, pleura, and pericardium, with distant pleural or pulmonary metastasis, but also to bone and liver.

The prognostic role of NLR in patients with thymic epithelial tumors has been investigated only in an extremely limited number of studies and has still to be completely assessed.

Yuan et al. [21] evaluated the value of NLR in 79 patients who underwent resection of thymic carcinoma over an 11-year period. According to the aggressive nature of the disease, a cut-off value of 4.1 was identified. High NLR resulted associated to tumor dimensions, Masaoka-Koga stage, worse DFS and OS. However, the marker did not result to be an independent prognostic factor of death or recurrence at multivariate analysis.

In 2017, Yanagiya et al. [22] analyzed preoperative NLR in 159 patients completely resected for thymoma between 1976 and 2015. Patients with NLR ≥1.96 had significantly shorter OS, recurrence-free survival, disease-specific survival, disease-related survival, and showed higher cumulative incidence of recurrence. Moreover, NLR resulted independently prognostic for recurrence in early-stage disease.

Recently, Janik et al. [23] reported about the prognostic value of NLR and other inflammatory markers in 122 patients affected by thymic epithelial tumors (75% thymoma, 25% thymic carcinoma). Higher preoperative values of NLR resulted predictive of lower freedom from recurrence at survival analysis, but not at multivariate analysis. Interestingly, this study included a longitudinal analysis of NLR variation on repeated measurements acquired during the follow-up, and association to the incidence of recurrence.

In our study, univariate survival analysis showed that, when the patients were stratified according to TNM stage, DFS was significantly lower in the group with higher NLR values. NLR could therefore be a useful tool to identify patients with surgically-treated thymoma at higher risk of relapse among the different stages of the disease. Nevertheless, with regard to locally-advanced disease, it is important to notice that such result may be conditioned by the small sample size of patients with TNM stage IIIA/B tumors.

A point which has to be taken into due consideration when selecting patients for adjuvant treatments is the fact that in the new TNM classification system for thymic malignancies [5] the percentage of patients with stage I disease is considerably higher (approximating to 80%) than that of other stages [6]. Local invasion of the mediastinal pleura is indeed considered to have negligible influence on prognosis [4], a point which causes downstaging of almost all previously Masaoka-Koga stage II as well as a significant number of stage III tumors, as confirmed by our study. However, further stratification of patients with early-stage disease seems advisable to select those patients who may benefit from adjuvant treatments in order to reduce the risk of recurrence [24].

Beyond the radiological assessment [25, 26], a number of markers, such as C-reactive protein, have been suggested as possible tools to improve the accuracy of follow-up [27]. If our results will be confirmed by larger studies, preoperative NLR could be used to identify patients with early stage thymoma at higher risk of relapse. Moreover, a few studies are currently in progress focusing on different steps of neutrophils-mediated cancer progression [7]. The introduction of new specific drugs may cover in the future a key role in targeted post-operative therapy of thymoma patients according to their NLR status.

The current indications to adjuvant therapy in early-stage thymoma are still a matter of debate. ESMO [25] and NCCN [26] guidelines recommend to consider post-operative radiotherapy in case of tumoral extension beyond the capsule, and state a clear indication in case of more invasive disease. Wu et al. [28] suggest irradiation following surgery for all Masaoka-Koga stages II and III, but advocate randomized clinical trials to assess its utility in stage I disease. The administration of fractioned radiotherapy with a total dose of 45 to 50 Gy is widely accepted, as it is able to reduce significantly the risk of recurrence. Moreover, the use of advanced techniques, such as intensity-modulated radiotherapy, is advocated to minimize the toxicity over the irradiated field [29].

Chemotherapy has seldom been adopted alone as adjuvant treatment in thymoma patients. Platinum-based regimens concomitant with radiotherapy are usually administered as first-line treatment [30]. Recently, Carillo et al. [31] demonstrated that adjuvant chemo-radiotherapy is able to improve survival in Masaoka-Koga stage II disease in case of WHO type B thymomas.

New parameters to identify early-stage tumors at higher risk of recurrence, such as NLR, could therefore be useful in the development of future strategies for adjuvant treatments.

The major limitations of this study are the retrospective design, the relatively small size of the cohort and the relatively short follow-up considering the indolent nature of the disease, which may present recurrences up to 10 years after surgery [2]. At multivariate analysis, NLR did not result an independent prognostic factor of relapse. In fact, other factors, such as the higher proportion of younger patients in the low-NLR-Group, may be responsible for their favorable DFS. Moreover, the limited number of TNM stage IIIA/B patients does not allow to establish significant conclusions about the prognostic role of NLR in locally-advanced thymoma. Further research with a multicenter prospective study is therefore needed to validate the use of this easily accessible and inexpensive tool in the selection process of candidates to adjuvant therapies and follow-up.

## Conclusions

Following the introduction of the 8th edition of TNM staging for thymic epithelial tumors, a large number of patients resected for thymoma will show early-stage disease. Therefore, further analysis to assess the risk of recurrence is essential.

Our analysis demonstrates that higher preoperative NLR seems to be associated to a worst outcome with regard to DFS in patients submitted to surgery for thymoma and stratified for TNM stage. In our opinion, this easy-available and cost-effective biomarker could therefore be of help for a better prognostic stratification among TNM stages, with particular notice to early-stage disease, to guide clinicians both for selection of candidates to adjuvant treatments and follow-up.

## Abbreviations

AUC: Area under curve
CI: Confidence interval
DFS: Disease-free survival
HR: Hazard ratio
IASLC: International Association for the Study of Lung Cancer
ITMIG: International Thymic Malignancies Interest Group
MG: Myasthenia gravis
MGFA: Myasthenia Gravis Foundation of America classification
NLR: Neutrophil-to-lymphocyte ratio
OS: Overall survival
PET: Positron emission tomography
ROC: Receiver-operating curve
TNM: Tumor – Node – Metastasis Staging System
VATS: Video-assisted thoracic surgery
WBC: White blood cell
WHO: World Health Organization
